# Evaluation of a flexible and integrative psychiatric care model in a department of child and adolescent psychiatry in Tübingen, Germany: study protocol (EVA_TIBAS)

**DOI:** 10.1186/s12913-021-07226-1

**Published:** 2021-11-22

**Authors:** Anne Neumann, Helene Hense, Fabian Baum, Roman Kliemt, Martin Seifert, Lorenz Harst, Denise Kubat, Birgit Maicher, Christopher Schrey, Jochen Schmitt, Andrea Pfennig, Ines Weinhold, Enno Swart, Bettina Soltmann

**Affiliations:** 1 Center for Evidence-Based Healthcare, University Hospital Carl Gustav Carus and Carl Gustav Carus Faculty of Medicine, Technische Universität Dresden, Fetscherstraße 74, 01307 Dresden, Germany; 2 WIG2 Scientific Institute for Health Economics and Health System Research, Leipzig, Germany; 3 Institute of Social Medicine and Health Services Research, Medical Faculty, Otto-von-Guericke- University Magdeburg, Magdeburg, Germany; 4 Department of Psychiatry and Psychotherapy, Carl Gustav Carus University Hospital, Technische Universität Dresden, Dresden, Germany

**Keywords:** Mental health care, Department of child and adolescent psychiatry, Flexible and integrated treatment, Evaluation, Cohort study, Qualitative and quantitative, Effectiveness and cost-effectiveness, Inpatient and outpatient treatment, Health services research, FIT

## Abstract

**Background:**

Model projects for flexible and integrated treatment (FIT) in Germany aim at advancing the quality of care for people with mental disorders. A new FIT model project was established in 2017 at the Department of child and adolescent psychiatry (KJP) of the University Hospital Tübingen (Universitätsklinikum Tübingen, UKT). The study design of EVA_TIBAS presented here describes the evaluation of the FIT model project at the KJP of the UKT. This evaluation aims at quantifying the anticipated FIT model project changes, which are to improve patients’ cross-sectoral care at the same maximum cost as standard care.

**Methods:**

EVA_TIBAS is a controlled cohort study using a mix of quantitative and qualitative methods. The FIT evaluation consists of three modules. In Module A, anonymized claims data of a statutory health insurance fund will be used to compare outcomes (duration of inpatient and day care psychiatric treatment, inpatient and day care psychiatric length of stay, outpatient psychiatric treatment in hospital, inpatient hospital readmission, emergency admission rate, direct medical costs) of patients treated in the model hospital with patients treated in structurally comparable control hospitals (estimated sample size = ca. 600 patients). In Module B, patient-reported outcomes (health related quality of life, symptom burden, return to psychosocial relationships (e.g. school, friends, hobbies), treatment satisfaction, societal costs) will be assessed quantitatively using validated questionnaires for the model and two control hospitals (estimated sample size = ca. 300 patients). A subsequent health economic evaluation will be based on cost-effectiveness analyses from both the insurance fund’s and the societal perspective. In Module C, about 30 semi-structured interviews will examine the quality of offer, effects and benefits of the service offered by the social service of the AOK Baden-Württemberg (for stabilizing the overall situation of care in the family) in the model hospital. A focus group discussion will address the quality of cooperation between employees of the university hospital and the social services.

**Discussion:**

The results of this evaluation will be used to inform policy makers whether this FIT model project or aspects of it should be implemented into standard care.

**Trial registration:**

This study was registered at ClinicalTrials.gov PRS (ID: NCT04727359, date: 27 January 2021).

## Background

According to the German KiGGS-Survey [1], about 20% of all children and adolescents in Germany are affected by mental and behavioral problems [2]. While about 6% of those under the age of 18 years are mentally ill according to diagnostic criteria and, as a consequence, are in need of treatment, about 50% of those in need do not receive treatment [3, 4]. Reasons for this poor claim of medical services among young patients might be low or delayed help-seeking behavior due to stigma, insufficient knowledge about access to mental health care services and lack of treatment capacities [5, 6]. Moreover, most adult mental health disorders have their onset in adolescence [7, 8]. These circumstances put further emphasis on the need of early prevention and adequate treatment of the young.

The treatment of patients with mental disorders in Germany is mainly provided by outpatient care, e.g. medical specialists, child and adolescent psychotherapists or psychiatric outpatient departments in hospitals (Psychiatrische Institutsambulanz (PIA)), the latter being for patients in need of particularly intensive and complex near-hospital care due to the nature, severity or duration of their mental disorder [3]. Mental health services are also provided in the inpatient sector, e.g. by hospitals with a Department of child and adolescent psychiatry (KJP). In addition, social service providers, such as the child and youth welfare service or educational and family counselling centers as well as the schools, e.g. school psychology services or schools for educational aid, support child and adolescent mental care [3]. Many other services, e.g. assisted living, social assistance or vocational reintegration, are also strongly involved in the care of patients with mental disorders.

The number of children treated in hospitals and diagnosed with a mental disorder has increased in Germany over the last years (2007–2014: + 38%) [3, 5], while the average duration of stay has decreased (2007–2014: − 14%) and at the same time, emergency rates have increased (2011: 29% vs. 2013: 38%) [3, 9, 10]. This situation is partly driven by a fraction of young patients being hospitalized with a high severity and complexity of psychosocial problems [9].

KJPs in Germany usually treat young patients under the age of 18 years. This means that treatment of patients in a KJP reaching the age of 18 years needs to be transferred to adult psychiatry. Thus, the entry into adulthood is a fragile period for those patients. Considering the care of young patients with mental disorders, further non-medical social service providers, such as schools, day care centers, youth welfare offices and the family system itself, need to cooperate closely with the health care providers. An interdisciplinary approach to care thus constitutes the core of the treatment in KJPs, including its implementation into the environment of the young patient, involving the custodians of the child or adolescent, who, in most cases, are the parents, [3].

The financing and operation of the German health care system is separated into sectors, each of which involves different legal grounds and stakeholders. For example, the remuneration of inpatient treatment differs from the one of outpatient treatment in the hospital and again from the remuneration of resident physicians, leading to a fragmented system with sometimes opposing interests of stakeholders [11]. The social and educational sectors are again organized and financed by different parties. Altogether, this strict separation of sectors in terms of regulatory frameworks, financing and remuneration hampers a patient-oriented management of psychiatric care. Treatment continuity is, however, particularly important for patients with mental disorders. The current strict separation of the financing hinders needs-adapted treatment forms as alternative treatment, such as intensive day care treatment or individual home care treatment, and are often not financially covered leading to more-than-necessary inpatient treatment. There is an ongoing debate in Germany on how to solve the problem of fragmentation at the federal level [12]. Several initiatives aim to reduce the fragmentation in the German health care system, e.g. projects for integrated or cross-sectoral care based on § 64b or 140a of the German Social Code, Book V (SGB V) [13, 14].

The introduction of § 64b SGB V in 2012 created the possibility of setting up FIT model projects for the advancement of the quality of care for people with mental disorders. The objective is to improve patient-oriented cross-sectoral care or optimize patient care. For this purpose, statutory health insurance (SHI) funds can close contracts with hospitals and jointly establish new and exclusive care and financing structures, forming the core of FIT model projects. The aim of the legislature was to establish these models, including KJP, in all 16 German federal states. By 2021, these goals have not yet been achieved. FIT models have only been established in 12 of the 16 federal states so far and only 7 of 22 FIT models have addressed children and adolescents. All FIT model projects must be scientifically and independently evaluated according to a supplementary law (§ 65 SGB V) [15].

Two German SHI funds (AOK Baden-Württemberg and the SVLFG, an agricultural health insurance fund)), have, together with the UKT, developed a FIT model project in the hospital’s Department of child and adolescent psychiatry, which will be tested for a total of 8 years starting from October 2017. This FIT model project is the only FIT model so far that exclusively targets children and adolescent at a KJP. Roughly 50% of patients treated in the KJP are insured through one of the two contracting SHI funds. The core of the model constitutes the Therapeutic Intensive Treatment in the Outpatient Setting (TIBAS, acronym for the German phrase “**T**herapeutische **I**ntensiv**b**ehandlung im **A**mbulanten **S**etting”), an approach that aims to provide more cross-sectoral services through flexible treatment intensities compared to standard care.

TIBAS as an intensive form of outpatient treatment intends to enable earlier discharge from the inpatient setting to improve psychosocial functioning by allowing patients to return into their social environment in a more timely manner. Different TIBAS levels have been implemented, representing different treatment intensities and frequencies, which can be deployed flexibly according to patients’ needs. A person-centered case manager accompanies patients throughout the whole treatment period. If necessary, patients and especially family members are accompanied by the social service of the AOK Baden-Württemberg and supported with regard to stabilizing the overall situation of care (psychosocial counseling and networking with other care providers, etc.). The social service supports custodians or relatives in unstable care situations. It follows a systemic approach that is based on the assumption that a sustainable stabilization of the child or adolescent can only be achieved through the stabilization of the custodians or relatives. The FIT model project at the UKT is, together with all other FIT projects at hospitals so far, based on a global treatment budget (i.e. fixed annual budget for all treated patients including inpatient care, day care and outpatient care [16]), but supplemented by additional revenue compensation. The focus of this model project is, however, based on the model specific element TIBAS, which is remunerated via per diem rates.

The study design presented here describes the evaluation of the FIT model project at the KJP of the UKT (EVA_TIBAS). The aim of the evaluation is to examine the achievement of the objectives of the FIT model project and to assess the transferability of the care approach into standard care. The primary objectives of the model project are

- to shorten the duration of inpatient stays while intensifying outpatient forms of treatment,
- to reduce the cumulative psychiatric treatment duration and the emergency admission rate,
- to reduce the symptom burden, to accelerate the return to psychosocial living conditions and
- to increase the quality of life.

The primary outcome measures for this evaluation are the change in inpatient psychiatric treatment and in quality of life. Stabilization of the family’s overall care situation will be used to sustain these improvements. Using qualitative research methods, the experiences with and the subjective benefit of the social service of the AOK Baden-Württemberg as well as the cooperation of this social service with the UKT will be examined in more detail. In addition, the costs of the FIT model care should not exceed those of the standard care or, at a maximum of the same costs, better results should be achieved with regard to the above-mentioned patient-related goals.

The EVA_TIBAS evaluation study will examine the FIT model project’s effectiveness in terms of treatment and patient-related outcomes, direct medical and indirect costs, and cost-effectiveness of the model compared to standard care.

## Methods/design

### Overall design

#### General

EVA_TIBAS is a controlled cohort study using a mix of quantitative and qualitative methods. The evaluation consists of three modules. In Module A, anonymized SHI claims data will be used to compare patients treated in the model hospital, i.e. KJP at the UKT, where the above described FIT model project is implemented and under evaluation (intervention group (IG)), with patients treated in control hospitals, i.e. structurally comparable hospitals from the federal state of Baden Württemberg (control group (CG)). In Module B, patient-reported outcomes will be quantitatively assessed using validated questionnaires in the model and two control hospitals. In Module C, semi-structured interviews and a focus group discussion will examine the additional offer of the social service of the AOK Baden-Württemberg in the IG (Table 1).

**Table.1:** Overview study design modules

Module	design	Intervention Group	Control group	time frame	data	outcomes
A	Cohort study, retrolective	Patients treated between Jan 2018 and Dec 2021 in the KJP of the UKT and insured with the SHI fund AOK Baden-Württemberg; *n* = ca. 600	Matched patients from up to 10 control hospitals treated between Jan 2018 and Dec 2021	Two years before and one to four years after reference date	Anonymized data from SHI fund AOK Baden-Württemberg	Duration of inpatient and day care psychiatric treatment, inpatient and day care psychiatric length of stay, outpatient psychiatric treatment in hospital, inpatient hospital readmission, emergency admission rate, direct medical care costs, and cost-effectiveness
B	Cohort study, prospective	Patients of the KJP of the UKT, insured with the SHI funds AOK Baden-Württemberg or SVLFG and treated within 18-month recruitment phase starting autumn 2021 (baseline); *n* = ca. 300	Patients from two structurally comparable hospitals (control hospitals) treated within 18-month recruitment phase starting autumn 2021	Baseline starting autumn 2021 and follow-up with 24 months after recruitment	patient-reported quantitative data	Health-related quality of life, symptom burden, return to psychosocial relationships, treatment satisfaction, societal costs, and cost-effectiveness
C	Cross-sectional					
C1	Semi-structured Interviews	Families of patients being treated in the KJP of the UKT, insured with the SHI fund AOK Baden-Württemberg and treated within 18-month recruitment phase starting autumn 2021 and with social service recommendation; *n* = ca. 30	NA	Between autumn 2021 and spring 2023	Personal telephone interviews	Offer and effects of social service, benefit from social service
C2	Focus group	UKT & social service personnel	NA	Spring 2022	Focus group discussion	Cooperation between UKT and social service employees

*KJP* Department of child and adolescent psychiatry, *UKT* University Hospital Tübingen, *SHI* Statutory health insurance, *reference date* First treatment in either intervention or control hospitals between 01 January 2018 and 31 December 2021, *n* Anticipated number of study participants in each module; *NA* Not applicable

**Fig. 1:**
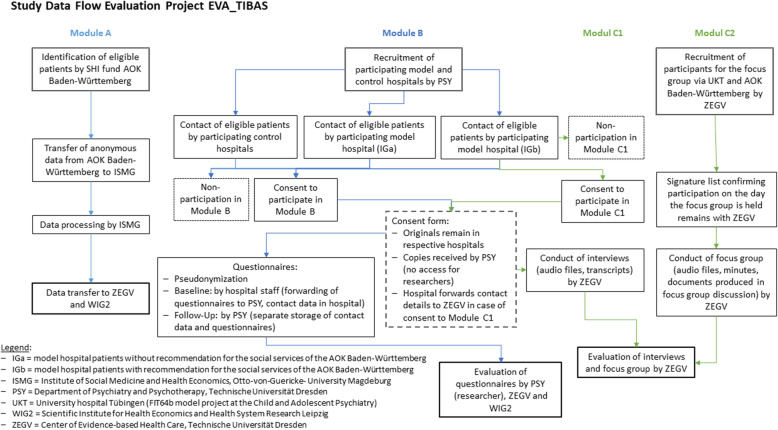
Study data flow chart

In order to ensure that potential outcome-related differences between IG and CG can be attributed solely to the intervention, both groups have to be as similar as possible to each other regarding all other aspects. Group comparability will be established in a two-step procedure. First, structurally comparable control hospitals were defined (see “selection of control hospitals” below). Second, from those identified control hospitals appropriate, comparable patients must be selected (see module descriptions). Further explanations of each module are given below. Figure 1 visualizes the study data flow in each of the modules.

The study team is supported by an advisory board with representatives from the German Society for Child and Adolescent Psychiatry, Psychosomatics and Psychotherapy (DGKJP) and the Federal Association of Relatives of Mentally Ill People e.V. (BApK), who give advice regarding study initiation, implementation and result interpretation.

### Outcomes and hypotheses

**Fig. 2:**
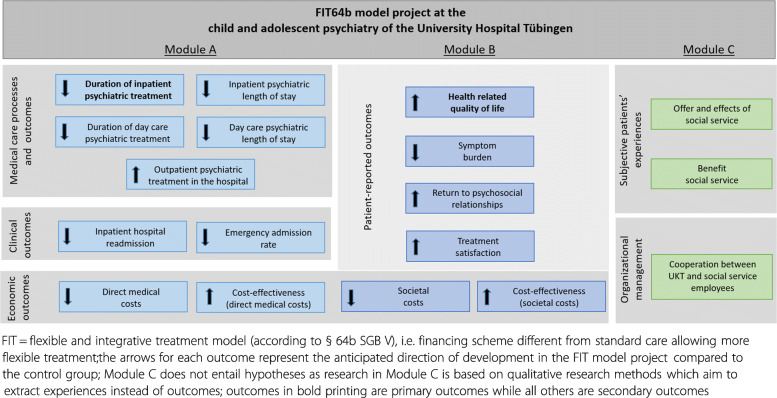
Outcomes and Hypotheses

Each module targets different research questions and underlying hypotheses (Fig. 2).

#### Primary outcome measures

- Duration of inpatient psychiatric treatment (Module A), i.e. the average cumulative inpatient psychiatric treatment duration (based on all included patients)
- Health-related quality of life (Module B), i.e. self-rated quality of life

#### Secondary outcome measures

- Duration of day care psychiatric treatment (Module A), i.e. the average cumulative day care psychiatric treatment duration (based on all included patients)
- Inpatient psychiatric length of stay (Module A) (based only on cases with inpatient psychiatric treatment)
- Day care psychiatric length of stay (Module A) (based only on cases with day care psychiatric treatment)
- Outpatient psychiatric treatment in the hospital (Module A), i.e. the intensity of outpatient psychiatric treatment in the hospital (PIA or TIBAS)
- Inpatient hospital readmission (Module A), i.e. percentage of patients with at least one second inpatient hospital stay within 12 months after discharge from first inpatient hospital stay
- Emergency admission rate (Module A), i.e. the percentage of patients with any psychiatric diagnosis (ICD-10: Fx) and emergency admission relative to all inpatient admissions with any psychiatric diagnosis within the respective reference hospital (either IG or CG)
- Direct medical care costs (Module A), i.e. the direct medical care costs of the psychiatric services from an SHI perspective
- Cost-effectiveness (direct medical costs) (Module A), i.e. comparison between duration of inpatient psychiatric treatment and direct medical care costs (SHI perspective)
- Symptom burden (Module B), i.e. measure of emotional symptoms, conduct problems, hyperactivity/inattention, peer relationship problems, and pro-social behavior
- Return to psychosocial relationships (Module B), i.e. measure of social integration (friends, school, sports activity, hobbies, social inclusion, day structure)
- Treatment satisfaction (Module B), i.e. self-perceived satisfaction with treatment
- Societal costs (Module B), i.e. costs from a societal perspective including additional costs not covered by the SHI funds, patients’ and relatives’ care, and travel expenses
- Cost-effectiveness (societal costs) (Module B), i.e. comparison between health related quality of life and societal costs (societal perspective)
- Offer and effects of social service (Module C), i.e. how families (with and without utilization of the social service) assess the social service offered by the AOK Baden-Württemberg and which additional effects do they experience using this service
- Benefit social service (Module C), i.e. to what extent do the users see a benefit in the advisory support provided by the social service
- Cooperation between UKT and social service employees (Module C) i.e. how the professional representatives of the UKT assess the cooperation with the social service of the AOK Baden-Württemberg and which strengths and optimization potentials are mentioned

### Selection of control hospitals

Structural deviations between model and potential control hospitals (similarity scores) were determined on the basis of structured quality reports according to § 137 SGB V [17] from the year 2016 (published in 2018) and matched data from the Federal Institute for Research on Building, Urban Affairs and Spatial Development (BBSR) [18], which included sociodemographic and socioeconomic data on the level of administrative districts (LANDKREISE) from the year 2016. Since the BBSR data from 2016 no longer included the number of psychotherapists and large differences in this parameter between the districts became obvious, the number of psychotherapists from the 2012 data was used instead. As the number of psychotherapists per administrative districts varied strongly over the past years, we will also examine the number of psychotherapists based on the demand planning / publication of the Association of statutory health insurance physicians [19] and include this information in the control hospital selection described above. Both lists will be compared before analyses for the interim report in Module A.

Based on these data, mandatory (i.e. hospital must have a PIA and be located in the Federal state Baden-Württemberg), hard (weighting: 50%, i.e. patients’ diagnoses) and soft criteria (25%, i.e. structural characteristics of the clinics, e.g. number of beds or personnel), and sociodemographic criteria of the region (25%, e.g. unemployment rate or average household income per inhabitant) were combined and a weighted similarity score to the model hospital was calculated.

Further details of this procedure are described elsewhere [20] and already used in other studies [21, 22]. All 16 possible control hospitals were sorted from best to least fit to the model hospital. For Module A, the ten best fitting hospitals will be selected for further consideration. The remaining possible control hospitals may be selected later in the study if chosen control hospitals merge, close or turn into a FIT model hospital. For Module B, possible control hospitals, according to the above-mentioned list, were consecutively asked to participate in the study until two control hospitals agreed to participate.

### Module A

#### Design

Module A describes the resource utilization in the model hospital including the shift from inpatient to outpatient treatment in the hospital, clinical outcomes and direct medical costs. Anonymous claims data from the SHI fund AOK Baden-Württemberg will be analyzed. Results will be separately analyzed between patients who have not been treated in the reference hospital, either model or control hospital, during the 2 years before study entry (hospital-new patients) and those who were treated at least once in the hospital in the 2 years before study entry (hospital-known patients).

All patients treated in the KJP at the UKT between January 2018 and December 2021 on an inpatient or outpatient basis, insured with the SHI fund AOK Baden-Württemberg and with follow-up data of at least 12 months after reference treatment will be included in the analysis for the IG. Due to the low number of those insured with the SHI fund SVLFG, this SHI will not be included in this analysis. The first 3 months after model project start (October – December 2017) will not be considered in this analysis due to initially incomplete implementation of model structures and benefit from consideration of complete calendar years. Patients selected via PIA treatment must be younger than 18 years of age at the time of the reference treatment.

Patients for the CG will be matched with patients from the above-described selected control hospitals. For each patient in the IG, one control patient will be matched from the patient pool of the selected control hospitals. For this purpose, two linked two-stage or mixed propensity score matches will be performed. The final decision about the consideration of patients for the analysis will be made using initial available data (power simulation).

The reference case refers to the initial treatment of the patient in either IG or CG after study onset, i.e. inclusion into the study in Module A. The reference date (Module A) refers to the date of the reference case. Outcomes in Module A will be compared to patients’ results 1 year prior to the reference case (pre-period) to better estimate the effect of FIT treatment.

#### Analysis

For each included patient, data starting from the reference date to at least 12 months and at most 48 months plus 24 months prior to reference date will be considered. Each outcome in this module, besides inpatient hospital readmission, will be compared between IG and CG and between the year prior to reference date and time after reference date (difference-in-difference approach). This approach enables the research team to observe changes in the outcomes under investigation over time and between IG and CG. The inclusion of control clinics allows the research team to mitigate the influence of unobservable exogenous effects (e.g., political or economic events) that may arise over time and affect both model and control clinics. This approach is successfully implemented in the EVA64 study [16, 21, 23].

A sample size of about 240 patients within 1 year with 120 patients in the IG and 120 patients in the CG and about 60 patient as hospital-new and 60 as hospital-known in each group is estimated. For the final report, about 600 patients are expected to be included in the dataset, i.e. 300 patients in each group, comprised of 60 hospital-new patients (4*60 patients) each year and 60 hospital-known patients. The anticipated number of patients per year is based on experiences in the IG and the relation between hospital-new and hospital-known patients on previous experiences of similar analyses [24]. An a priori power analysis for the primary outcome parameters using α = 0.05 and 1 – β = 0.80 revealed that medium effects (Cohen’s d = 0.5) could be verified with the estimated dataset.

Generalized regression models with additive modeling of the treatment effect will be used. Comparisons between IG and CG will be modeled for each measurement point (one measurement point per patient-specific year), each in relation to the pre-period. The outcome inpatient readmission is not suitable for a longitudinal analysis due to its operationalization. Therefore, the inferential statistical evaluation here will exclusively consider the first patient-specific year. Whenever possible, the models will be adjusted for confounder variables, i.e., other available influencing variables (e.g. age group, gender, comorbidity, setting of reference case) are included in the model to control for additional variance explanation of these variables.

Effects and costs will be analyzed from a SHI perspective. Primary (confirmatory) analyses will be performed according to the intention-to-treat approach firmly assigning patients to the hospital that initially filed their reference case. For the cost-effectiveness analysis, primary outcome parameters and costs will be compared between model and control hospitals using the incremental cost-effectiveness ratio as costs per one-day-of-hospital-stay avoided. A significance level of *p* ≤ 0.05 will be set for all analyses.

### Module B

#### Design

In Module B, the KJP departments of the selected control hospitals will be asked for study participation based on the above described ranked list of identified control hospitals. Recruitment of control hospitals will start with the hospital best fitting the model and continued down in ranking until at least two control hospitals will have agreed to participate. After initiation, all patients being treated in the participating hospitals within an 18-month recruitment phase starting in spring 2021 will be asked for study participation. As the targeted study population is underage, custodians, together with the patient, will be asked for informed consent regarding study participation. Custodians and siblings of the patients will also be recruited and asked for study participation and informed consent. Having given informed consent, patients, custodians and siblings will be asked by the hospital staff to complete a set of questionnaires (Table 2) (t = 0). Age-appropriate versions of the questionnaires (group of 6–7 year olds, 8–10 year olds, and 11–17 year olds) will be used for the children and adolescents. Follow-up will occur after 24 months with the same set of questionnaires used during baseline (t1).

**Table.2:** Instruments Module B

Outcomes	Subject^a^	user^b^	ITEMS/QUESTIONNAIRES
basic data on socio-demographics	patient	patient	Age, sex, type of school, grade
		custodian	Year of onset of disease, number of former hospital stays
Disease		Staff at participating hospital	Psychiatric and somatic diagnoses (ICD-10), date of admission and discharge, setting, patient’s placement
health-related quality of life		patient	Health questionnaire KIDSCREEN-27 [25]
		custodian	Health questionnaire KIDSCREEN-27, parent version [25]
Symptom burden		patient & custodian^c^	Strengths and Difficulties Questionnaire (SDQ) [26]
Return to psychosocial relationships		patient & custodian	Own questions (friends, school, physical activity, hobbies, social integration, day structure)
treatment satisfaction		patient & custodian^c^	Treatment assessment questionnaire (FBB) [27]
costs		custodian	Own questionnaire adapted from the Client Sociodemographic Service and Receipt Inventory (CSSRI) [28] and the “Questionnaire on the use of medical and non-medical care services for mental illnesses” (FIMPsy) [29]
Burden on relatives	custodian	custodian	Questionnaire on the burden of relatives (FBA) [30]
	siblings	siblings	Health questionnaire KIDSCREEN-27 [25] for siblings Questionnaire on the burden of siblings (LARES) [31]

^a^Subject = individual about whom outcomes are measured

^b^User = individuals who fill in the questionnaire (on behalf)

^c^depends on the age of the participating patient

A nonparticipant survey will be conducted in which the reasons for nonparticipation as well as a minimum set of sociodemographic and morbidity-related data of the patients will be recorded to compare such characteristics of the study nonparticipants with those of participating patients to estimate the extent of possible selection bias. Patients asked for participation in this module must be at least 6 years old, must have sufficient cognitive and linguistic abilities to participate in the survey, and must have provided written consent (of the children and adolescents themselves plus written consent of their custodians). Patients posing an acute danger to themselves and others will not be allowed to participate in this study.

The questions on the return to psychosocial living conditions will be validated internally before use. Furthermore, a pre-test of the questionnaires (see Table 2) will be conducted. For this purpose, the questionnaires will be tested in their age-appropriate versions on at least ten children or adolescents of the respective age group (6–7, 8–10, 11–17 years) for usability (manageability & comprehension), acceptability (number of items) and faking (response tendencies). Necessary changes revealed in this pre-test will be incorporated in the final version of the questionnaires.

#### Analysis

An evaluation of the characteristics of the IG and CG, e.g. age, gender, diagnosis, etc., will be performed prior to the evaluation of the hypotheses under investigation. Patient matching via entropy balancing will be employed in this module to achieve higher power in the analysis as well as higher representativeness as a loss of subjects due to extreme covariate values is minimized with this balancing method [32–35].

It is anticipated to have about 100 patients recruited in each participating hospital, i.e. 100 in IG and 200 in CG, over the 18-month recruitment phase. Those patients will be contacted in the follow-up after 24 months. We anticipate a dropout rate of .50, based on the response rate of previously conducted studies with comparable study design. Using α = 0.05 and 1 – β = 0.80, medium effects (Cohen’s d = 0.5) could be verified within this module.

The primary outcome, health related-quality of life, will be assessed confirmatory for subgroups regarding differences between IG and CG. T-test analyses will be used to compare different groups and the analysis of covariance will be used to adjust for baseline differences and other intervening covariates. A significance level of *p* ≤ 0.05 will be set for all analyses.

### Module C

#### Design

In Module C, individual experiences with the social service of the AOK Baden-Württemberg will be assessed among the families, the employees of the UKT and the social service of the AOK Baden-Württemberg using semi-structured interviews and a focus group discussion. The evaluation of the social service of the AOK Baden-Württemberg consists of two parts:

1. Semi-structured interviews with custodians of the participating children and adolescents and
2. A focus group discussion with staff of the UKT and the social service

1. Semi-structured interviews (C1)

The research aim is to understand how the social service supports the families, why families use or do not use the service and how the offer and service could be improved. Families of the IG with a recommendation to refer to the counseling support of the social service of the AOK Baden-Württemberg will be interviewed by telephone using semi-structured interview guidelines. Eligible family members will be recruited via the recruitment procedure of study participants in Module B. The staff of the IG knows whether the social service had been offered to the family of the recruited patient. Families with a recommendation for the social service will be asked to participate in Module C (C1). Prerequisites for participation in Module C1 are sufficient knowledge of the German language, cognitive and linguistic abilities to participate in the interviews and written informed consent of the family members.

Family members who have used the social service of the AOK Baden-Württemberg will be asked

- how they have perceived the offer of the social service,
- which assistance measures they have utilized,
- whether and how the offered help has supported the family in dealing with their offspring’s condition,
- which measures taken by the social service have specifically proven helpful or not helpful from their point of view, and
- which services should to be offered continuously in the future.

Furthermore, family members who had not used the social service will be asked why and especially, which barriers made the participation more difficult and which further support they would wish for. Interview guideline construction is guided by the SPSS-principle (brainstorming, proofing, sorting, subsumption) by Helfferich (2011) [36]. Questions will be formulated in an open-ended manner. To reduce suggestiveness the interview guideline will be developed and discussed in a group of four scientists (psychology and social science).

2) Focus group discussion (C2)

The focus group will be composed of employees of the UKT who are involved in FIT implementation, and employees of the social service of the AOK Baden-Württemberg. The purpose of this focus group is to evaluate the cooperation between the hospital and the social service of the AOK Baden-Württemberg and to identify strengths and potential for improvement. Participants in the focus group should have several months of experience in cooperation with the social service of the AOK Baden-Württemberg or the staff of the UKT within the frame of the FIT model project. Currently (September 2021), five case managers, one current and one former social worker fulfill these criteria and will be invited for the focus group discussion in spring 2022 (*n* = 7). The focus group interview guideline will be developed in a group of four scientists (psychology and social science) based on a model of interdisciplinary collaboration by Bronstein (2003) [37] and an investigation of Gabrielova & Velemínský (2014) [38] about interdisciplinary collaboration between medical and non-medical professions in health and social care.

#### Analysis

During the 18-month recruitment phase for the interviews (C1), about 30 interviews are expected to be conducted including those who used the social service of the AOK Baden-Württemberg and those who did not. The number of 30 interviews is an estimation: In 18 months, about 150 families receive a recommendation for the social service. All of them will be asked, if they want to participate in an interview. We expect that around 25% of all families with recommendation will contact the social service (*n* = 38). If half of them agree to an interview, 19 persons with use of the social service can be interviewed. For the 112 families without use of the social service despite recommendation, the agreement rate for an interview is estimated to be lower. If 20% of them agree for an interview, 22 persons can be interviewed. Anticipated data saturation [39] is expected for 30 interviews. Recruitment ends when 30 people have been interviewed or when the recruitment period of 18 months ends.

The interviews and focus group discussion will be conducted by experienced interviewers or focus group leaders with knowledge of qualitative social research methods. If the study participants agree, the guided interviews and focus group will be recorded using a tape recorder. In addition, the interviews will be simultaneously recorded in written form. In the focus group discussion, jointly created charts (e.g., map query) and prioritizations will document the results. The evaluation and analysis of the written data will be carried out according to the qualitative content analysis based on Mayring (2010), following both the deductive and the inductive process of category formation [40].

## Discussion

The evaluation of the effects, costs and cost-effectiveness of the FIT model hospital of the KJP at the UKT compared to standard care as well as the perceived offer, effects and benefits of and the cooperation with the social service is described in this manuscript. This study is rather a multi-methods study than a mixed-methods approach. Mixed-methods refers to the collection of different types of data (quantitative and qualitative) and their integration [41]. This integration should result in an added value which would not be present evaluating both parts separately [42]. The integration of qualitative and quantitative data (Module A or B with Module C) was not possible in this study due to data protection issues and differing research questions. However, the results of all three modules will be jointly interpreted.

### Strengths and limitations

The mix of claims data and primary data allows for a holistic evaluation of the model project. The strengths of the claims data, such as the absence of selection and recall bias, are combined with the strengths of primary data, such as consideration of patients’ preferences and experiences. Further, the selected control groups in Module A and B allow a comparison to standard care. In addition, the long time horizon of the evaluation allows consideration of up to 4 years follow-up time after reference treatment plus 2 years before evaluation inclusion (Module A) and a recruitment phase of 18 months (Module B and C) as well as a follow-up after 24 months (Module B). The huge array of SHI data covering information on inpatient, day care and outpatient utilization as well as cost items enables the researchers to assess comprehensively effects measured as inpatient and outpatient care and costs. Moreover, the study’s advisory board strengthens the perspective of those giving care to children and adolescents as well as the relatives of those affected.

Besides all the mentioned strengths, some limitations need to be considered as well. First, due to a limited target group at the UKT, i.e. only children and adolescents being treated at the Department of child and adolescent psychiatry of the UKT, a relatively small sample size is estimated. A small sample size might infer that small effects will not be detected. In addition, starting such an evaluation in the current SARS-CoV-2 pandemic might hinder adherence to the planed time schedule due to anticipated delay in ethics vote and recruitment. Further, the probably altered utilization of health services due to the SARV-CoV-2 pandemic might hamper the true estimation of resource utilization. However, the control group will help in the estimation of their effects in the evaluation and will balance possible bias here. So far, we expect that the effect of the pandemic might affect IG and CG equivalently. Nonetheless, the potential effects of the pandemic on the model project will be closely investigated.

## Conclusions

The results of this evaluation will be used to inform policy makers whether this FIT model project or aspects of it should be included in standard care. Thus, it will not only benefit the participating parties, such as the SHI funds and the model hospitals, but it will also provide further insight into the improvement of the care of children and adolescents with mental disorders.

## Data Availability

The datasets generated and analyzed during the current study are not publicly available due to data protection and privacy reasons.

## Abbreviations

CG: Control group, i.e. patients from structurally comparable control hospitals
ICD-10: International Classification of Disease, 10th revision
IG: Intervention group, i.e. patients from the FIT hospital
PIA: Psychiatric outpatients department
SGB: Social Security Code (Sozialgesetzbuch)
